# Genome-wide linkage scan for factors of metabolic syndrome in a Chinese population

**DOI:** 10.1186/1471-2156-11-14

**Published:** 2010-02-24

**Authors:** Claudia HT Tam, Vincent KL Lam, Wing-Yee So, Ronald CW Ma, Juliana CN Chan, Maggie CY Ng

**Affiliations:** 1 Department of Medicine and Therapeutics, The Chinese University of Hong Kong, The Prince of Wales Hospital, Shatin, Hong Kong SAR, China; 2 Li Ka Shing Institute of Health Sciences, The Chinese University of Hong Kong, The Prince of Wales Hospital, Shatin, Hong Kong SAR, China; 3 Hong Kong Institute of Diabetes and Obesity, The Chinese University of Hong Kong, The Prince of Wales Hospital, Shatin, Hong Kong SAR, China; 4 Current Address: Department of Pediatrics, Section on Medical Genetics, and Centers for Human Genomics and Diabetes Research, Wake Forest University School of Medicine, NC, USA

## Abstract

**Background:**

Shared genetic factors may contribute to the phenotypic clustering of different components of the metabolic syndrome (MES). This study aims to identify genetic loci that contribute to individual or multiple factors related to MES.

**Results:**

We studied 478 normoglycemic subjects ascertained through 163 families participating in the Hong Kong Family Diabetes Study. Factor analysis on 15 MES-related traits yielded 6 factors including adiposity factor (body mass index, waist and hip circumferences), insulin factor (fasting insulin and insulin AUC during OGTT), glucose factor (fasting glucose and glucose AUC during OGTT), TC-LDLC factor (total cholesterol and LDL-cholesterol), blood pressure factor (systolic and diastolic blood pressure) and TG-HDLC factor (triglycerides and HDL-cholesterol). Genome-wide linkage analyses were performed on these factors using variance component approach. Suggestive evidence for linkage (LOD = 1.24 - 2.46) were observed for adiposity factor (chromosome 1 at 187 cM, chromosome 9 at 34 cM and chromosome 17 at 10 cM), insulin factor (chromosome 2 at 128 cM, chromosome 5 at 21 cM and chromosome 12 at 7 cM), glucose factor (chromosome 7 at 155 cM), TC-LDLC factor (chromosome 7 at 151 cM and chromosome 13 at 15 cM) and TG-HDLC factor (chromosome 7 at 155 cM).

**Conclusions:**

In summary, our findings suggest the presence of susceptibility loci that influence either single (chromosomes 1, 2, 5, 9, 12, 13 and 17) or multiple factors (chromosome 7) for MES in Hong Kong Chinese without diabetes.

## Background

Metabolic syndrome (MES) represents a clustering of multiple metabolic abnormalities, including insulin resistance, glucose intolerance, hyperinsulinemia, dyslipidemia, hypertension, and obesity [1,2]. These metabolic factors interact to substantially increase the risk of cardiovascular diseases, chronic kidney disease, and type 2 diabetes (T2D) [3-5]. Similar to figures reported in the US, [6], the prevalence of MES ranges from 10% to 30% in Asian populations [7]. In Hong Kong, where 95% or more of the residents are Han Chinese, the prevalence of MES was 17% in 2000 [8]. After 6 years of follow up, subjects with MES had 4-5 fold increased risk of new onset of diabetes [9] and 2-3 fold increased risk of heart disease and all-cause mortality [10].

Principal component factor analysis (PCFA) is a multivariate statistical technique used to extract a set of latent or underlying independent factors from a large number of inter-correlated variables. Edwards et al. [11] firstly used factor analysis to understand the underlying relationships among metabolic risk variables. In the past decade, we [12,13] and others have reported the composition of multiple factors and their overlapping nature in subjects with MES [14-18].

In a working population of Hong Kong Chinese, we used structural equation modelling and identified age, family history and obesity as the major explanatory variables for all components of MES including insulin resistance [13]. In the Hong Kong Family Diabetes Study (HKFDS), we further demonstrated that all metabolic traits of MES including adiposity, blood pressure, lipids, insulin resistance and beta-cell function were highly heritable (heritability = 0.45 - 0.63) [19]. Subsequent genome-wide linkage analyses of this family-based cohort revealed linkage at multiple chromosomal regions for diabetes, MES and/or related quantitative traits [20,21].

Previous studies in Caucasian populations have suggested that different factors of MES may be regulated by shared genetic factors which may partly explain the clustering of MES factors within an individual [22,23]. In this study, we applied PCFA to identify six independent factors of MES (adiposity, insulin, glucose, TC-LDLC, blood pressure and TG-HDLC factors). Then we tested for the hypothesis if shared or independent genetic factors contribute to these MES factors by performing genome-wide linkage analysis in 478 normoglycemic subjects from 163 families ascertained from the HKFDS.

## Results

The clinical characteristics of 478 normoglycemic family members in 163 families are summarized in Table 1. Table 2 shows the pairwise Pearson product moment correlation coefficients of the 15 MES-related quantitative traits. The adiposity traits were highly correlated with each other (r > 0.76). In addition, body mass index (BMI) and waist circumference (WC) showed moderate correlations with all other traits (r = 0.24 to 0.44). On the other hand, HDL-cholesterol (HDLC) showed moderate inverse correlations with other traits (r = -0.13 to -0.39), except for total cholesterol (TC).

**Table 1:** Clinical characteristics of normoglycemic first degree relatives recruited from 163 families of probands with diabetes.

Characteristics	Value
*N*	478
Male/female	199/279
Age (years )	37 ± 13
Body mass index (kg/m^2^)	23.8 ± 4.1
Waist circumference (cm)	78 ± 10
Hip circumference (cm)	96 ± 8
Fasting Insulin (pmol/l)	46 (43 - 48)
Insulin AUC 0 - 30 minutes (pmol/l)	6000 (5668 - 6352)
Insulin AUC 0 - 120 minutes (pmol/l)	38897 (36954 - 40964)
Fasting plasma glucose (mmol/l)	4.9 ± 0.5
Plasma glucose AUC 0 - 30 min (mmol/l)	202.1 ± 29.5
Plasma glucose AUC 0 - 120 min (mmol/l)	887.4 ± 199.4
Systolic blood pressure (mmHg)	120 ± 17
Diastolic blood pressure (mmHg)	73 ± 12
Total cholesterol (mmol/l)	5.0 ± 1.0
LDL cholesterol (mmol/l)	3.0 ± 0.9
Triglyceride (mmol/l)	1.01 (0.96 - 1.06)
HDL cholesterol (mmol/l)	1.4 ± 0.4

Data are shown as mean ± SD or geometric mean (95% CI). AUC, Area under curve

**Table 2:** Pearson product moment correlation coefficients of the 15 metabolic syndrome-related traits in normoglycemic family members.

Phenotype	1	2	3	4	5	6	7	8	9	10	11	12	13	14	15
**1. BMI**	**--**														
**2. WC**	0.854	**--**													
**3. HC**	0.825	0.776	**--**												
**4. Fasting PI**	0.404	0.319	0.307	**--**											
**5. PI AUC 0 - 30 min**	0.291	0.275	0.281	0.475	**--**										
**6. PI AUC 0 - 120 min**	0.364	0.348	0.295	0.530	0.717	**--**									
**7. Fasting PI**	0.353	0.419	0.253	0.174	-0.068	0.095	--								
**8. PG AUC 0 - 30 min**	0.266	0.347	0.143	0.143	0.184	0.241	0.617	**--**							
**9. PG AUC 0 - 120 min**	0.360	0.400	0.192	0.180	-0.014	0.327	0.576	0.718	**--**						
**10. SBP**	0.367	0.438	0.278	0.096	0.027	0.074	0.329	0.309	0.354	**--**					
**11. DBP**	0.386	0.419	0.246	0.190	0.099	0.130	0.234	0.241	0.288	0.741	**--**				
**12. TC**	0.242	0.306	0.141	0.023	-0.007	0.060	0.264	0.261	0.239	0.165	0.112	**--**			
**13. LDL**	0.276	0.354	0.200	0.015	-0.003	0.069	0.290	0.264	0.236	0.154	0.109	0.930	**--**		
**14. TG**	0.303	0.361	0.203	0.268	0.256	0.314	0.275	0.302	0.299	0.169	0.170	0.287	0.199	**--**	
**15. HDL**	-0.298	-0.391	-0.259	-0.169	-0.153	-0.250	-0.240	-0.173	-0.198	-0.139	-0.157	0.105	-0.131	-0.387	**--**

In the factor analysis of these 15 traits, six factors were extracted and cumulatively explained 82% of the total variance (Table 3). The six extracted factors are consistent with the inter-relationship among closely related traits (Table 2) and are interpreted as follows: Factor 1 refers to "adiposity factor", with high positive loadings of BMI, WC and hip circumference (HC); factor 2 refers to "insulin factor" with strong positive correlations with plasma insulin (PI) areas under the curve (AUC) for 0 - 30 min and PI AUC for 0 - 120 min and moderate positive correlation with fasting PI; factor 3 refers to "glucose factor" with high positive correlations among fasting plasma glucose (PG), PG AUC for 0 - 30 min and PG AUC for 0 - 120 min; factor 4 refers to "TC-LDLC factor", dominated by a high positive correlation between TC and LDL-cholesterol (LDLC); factor 5 refers to "BP factor", with large positive loadings for systolic and diastolic blood pressure (SBP and DBP); factor 6 refers to "TG-HDLC factor", with strong but inverse relationship between triglycerides (TG) and HDLC. All six factors showed moderate to high heritabilities, ranging from 29 to 67% (Table 3).

**Table 3:** Factor loadings and variance explained by the factors after varimax rotation in the PCFA model.

Phenotype/Interpretation	Factor 1	Factor 2	Factor 3	Factor 4	Factor 5	Factor 6
	Adiposity	Insulin	Glucose	TC-LDL	Blood Pressure	TG-HDL
**Body mass index (kg/m**^**2**^**) **^**a**^	**0.87**	0.24	0.15	0.10	0.16	-0.15
**Waist circumference (cm) **^**a**^	**0.87**	0.22	0.17	0.09	0.16	-0.19
**Hip circumference (cm) **^**a**^	**0.93**	0.13	0.06	0.06	0.10	-0.05
**Fasting Insulin (pmol/l) **^**a**^	0.23	**0.68**	0.09	0.03	0.17	-0.12
**Insulin AUC 0 - 30 minutes (pmol/l) **^**b**^	0.13	**0.90**	-0.03	-0.01	0.00	-0.02
**Insulin AUC 0 - 120 minutes (pmol/l) **^**b**^	0.16	**0.86**	0.21	0.02	0.03	-0.15
**Fasting plasma glucose (mmol/l) **^**a**^	0.23	-0.09	**0.76**	0.08	0.06	-0.13
**Plasma glucose AUC 0 - 30 min (mmol/l) **^**a**^	0.00	0.21	**0.87**	0.05	0.05	0.01
**Plasms glucose AUC 0 - 120 min (mmol/l) **^**a**^	0.10	0.12	**0.84**	0.03	0.12	-0.13
**Systolic blood pressure (mmHg) **^**a**^	0.19	0.00	0.15	-0.05	**0.88**	-0.03
**Diastolic blood pressure (mmHg) **^**a**^	0.12	0.15	0.04	-0.02	**0.91**	-0.06
**Total cholesterol (mmol/l) **^**a**^	0.05	0.03	0.06	**0.99**	-0.01	0.04
**LDL cholesterol (mmol/l) **^**b**^	0.13	0.00	0.07	**0.94**	-0.06	-0.08
**Triglyceride (mmol/l) **^**a**^	0.11	0.20	0.14	0.26	0.10	**-0.79**
**HDL cholesterol (mmol/l) **^**b**^	-0.18	-0.06	-0.09	0.16	0.00	**0.87**
	
**Eigenvalue**	4.72	1.99	1.73	1.57	1.27	1.09
**Total variance (%)**	31.45	13.29	11.55	10.44	8.46	7.28
**Cumulative variance (%)**	31.45	44.74	56.29	66.73	75.18	82.46
	
**Heritabilities**	61.69	55.62	28.58	67.03	64.15	64.00

^**a **^and ^**b **^indicates natural logarithm and square root transformation. Factor loadings > 0.4 are highlighted in boldface.

**Figure 1:**
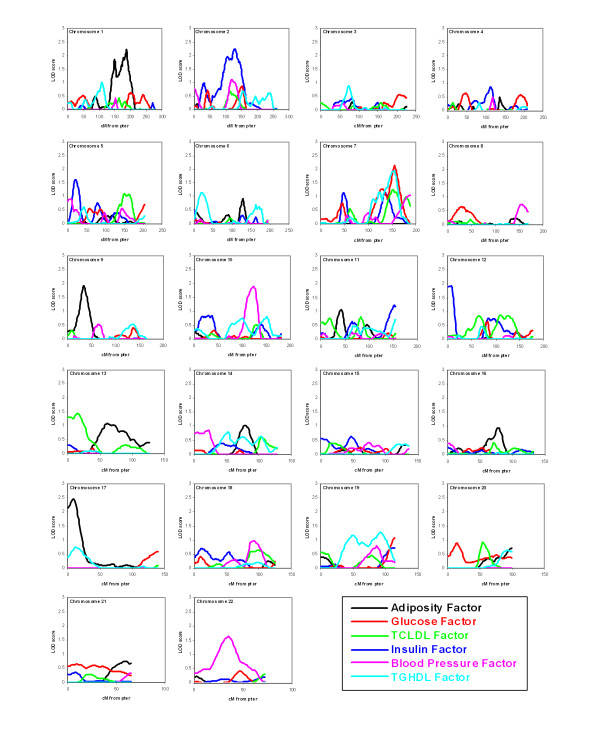
**Multipoint linkage analyses results of the six factors**. The horizontal axis is cM from p-terminus.

**Figure 2:**
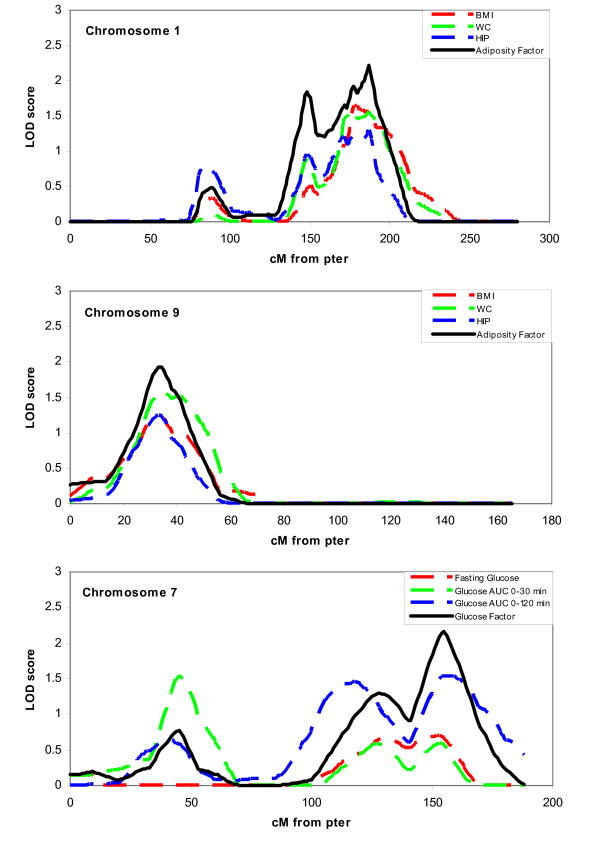
**Comparisons of multipoint linkage analyses results for metabolic factors and its individual components**. a) Adiposity factor vs BMI, WC and HC on chromosome 1. b) Adiposity factor vs BMI, WC and HIP on chromosome 9. c) Glucose factor vs fasting plasma glucose (PG), PG AUC 0-30 minutes and PG AUC 0-120 minutes on chromosome 7. The horizontal axis is cM from p-terminus.

In the variance component multipoint linkage analyses, we observed suggestive evidence of linkages (empirical p-value < 0.01) on chromosomes 1, 9 and 17 for adiposity factor (LOD = 1.92 - 2.46), chromosomes 2, 5 and 12 for insulin factor (LOD = 1.61 - 2.23), chromosome 7 for glucose factor (LOD = 2.16) and TG-HDLC factor (LOD = 1.96), as well as chromosomes 7 and 13 for TC-LDLC factor (LOD = 1.24 - 1.43) (Table 4 and Figure 1). Of note, the chromosome 7 region at 155 cM demonstrated a clustering of linkage signals for glucose, TC-LDLC and TG-HDLC factors. When the linkage results from PCFA were compared to that of their respective individual components (see Additional file 1), we observed consistent linkages for individual components for adiposity factor on chromosomes 1 and 9 and for glucose factor on chromosome 7, with the linkage signals from PCFA stronger than the individual components (Figure 2). On the other hand, the linkage signals for PCFA factors in other regions were modest compared to the varied linkage signals strength for individual components (see Additional file 1).

**Table 4:** Regions showing nominal evidence of multipoint linkage (empirical p-value < 0.01) to the factors.

Factors	Interpretation	Chr	**Position (cM) **^**a**^	Flanking markers	LOD	Empirical P-value
1	Adiposity	1	187	DIS466 - D1S202	2.22	0.0022
		9	34	GATA187D09 - D9S925	1.92	0.0038
		17	10	D17S1308 - D17S1298	2.46	0.0013
2	Insulin	2	128	D2S2972 - D2S1328	2.23	0.0018
		5	21	D5S2505 - D5S1486	1.61	0.0070
		12	7	D12S372	1.92	0.0037
3	Glucose	7	155	D7S1804 - GATA104	2.16	0.0003
4	TC-LDLC	7	151	D7S1804 - GATA104	1.24	0.0090
		13	15	D13S787 - ATA5A09	1.43	0.0064
6	TG-HDLC	7	155	D7S1804 - GATA104	1.96	0.0020

^**a **^Marker positions are indicated as cM from pter in the deCODE Map.

## Discussion

This genome scan analysis was performed in normoglycemic subjects in the HKFDS. The original study consists of 913 first degree relatives recruited from 179 families with predominantly young-onset diabetes. In this cohort, 53% of the probands and 25% of the siblings had MES. All metabolic traits of MES demonstrated high heritability estimates ranging from 0.45 to 0.63 [19]. In a linkage analysis based on 64 families with 126 affected sib-pairs segregating the diabetes trait, we identified suggestive linkage to chromosomes 1, 4, 6 and 12 [20]. In a follow up analysis using the entire family-based cohort, we reported suggestive linkage of MES traits to chromosomes 1, 2 and 16 [21].

To delineate whether the linkage signals observed in our previous linkage studies affect single or multiple metabolic factors, we genotyped additional microsatellite markers within these previously linked regions. Only subjects with normoglycemia were analyzed in the present study in order to avoid possible modifying effect of diabetes on the metabolic traits. Using PCFA, we identified six discrete factors loaded with closely related variables, namely adiposity, insulin, glucose, TC-HDLC, TC-LDLC and BP. Principal component factor analysis (PCFA) aimed to reduce a set of observable variables to a small number of latent factors that account for the correlations among observed variables. This analysis strategy compensates the problem associated with multiple comparisons and increases the power significantly [24]. For example, only one test of linkage analysis was applied on the adiposity factor in our study instead of three linkage tests on the corresponding individual quantitative traits including BMI, WC, and HIP.

Many of our linkage signals were linked to chromosomal regions observed by other genome scans for hypertension [25,26], fasting insulin [27,28], dyslipidemia [29], visceral fat area [30] and/or low metabolic rates [31]. Interestingly, several of these regions were also linked to obstructive sleep apnoea [32] which is closely associated with obesity. Additional file 2, table S2 summarized the linkage findings in the present and other previous studies.

Despite excluding subjects with diabetes, we found significant linkage of adiposity factor to chromosome 1q at the 187 cM region. The linkage of chromosome 1q 21-25 region to type 2 diabetes has been replicated in at least 8 independent populations suggesting that this region is likely to harbor major gene(s) for diabetes and/or MES [33]. The overlapping nature of this region linked to diabetes, MES and adiposity traits in our families suggests that genetic variants in this region may interact with other factors to give rise to diabetes. In addition, we found regions linked to adiposity on chromosomes 9 (34 cM) and 17 (10 cM). These regions were close to the linkage signals reported in other genome scans of MES or related traits. In the GIFT Study, a significant evidence for a region on chromosome 17 (29 - 58 cM) linked to T2D was reported [34]. This region contains a large number of genes which may influence various transcription and signaling pathways in energy metabolism. These include glucagon-like peptide 2 receptor (*GLP2R*), mitogen-activated protein kinases (*MAPK*) *MAP2K4 *and *MAP2K3*; transcription factors, (*STAT*)*5A *and *STAT5B*, neuropeptide pancreatic polypeptide 2 (*PPY2*); complement C1q receptor protein (*C1QBP*); lipoxygenase (LO) gene family, *ALOX12 *and *ALOX15 *[27]. The other regions on chromosome 9 linked to adiposity factor in our cohort were also significant in other genome scans for hypertension [26], familial dyslipidemia [29], visceral fat area [30] and HDLC [30,35] in other populations.

In this analysis, the insulin factor was linked to the 128 cM of chromosome 2. Interestingly, an adjacent region at 94-118 cM has been reported in several genome scans for hypertension in both Caucasian and African populations [26,36]. This region harbors a number of genes which regulate blood pressure and vascular reactivity such as the α2β adrenergic receptor gene, a key component of the sympathetic nervous system, *SCN7A *of the sodium channel and *NOSTRIN *which is related to the NO pathways [36].

The other region of interest is chromosome 5 at 21 cM which was also linked to the insulin factor. In a recent 2-dimensional genome scan for hypertension, novel epistatic loci was identified notably on chromosome 5q13.14 which interact with chromosomes 9, 11, 15, 16 and 19 [37]. In the Hypertension Genetic Epidemiology Network (HyperGEN) of the Family Blood Pressure Program, a genome scan also identified a linkage signal on chromosome 5 at 20 cM with a LOD score of 2.36 in European Americans. Both diabetes and hypertension are heterogeneous disorders with multiple and interacting aetiologies, including activation of stress hormonal systems, hyperinsulinemia, abnormal vascular smooth muscle cellular growth and sodium and water retention [38]. To this end, subjects with genetic predisposition to hyperinsulinemia are prone to develop hypertension, which may be accelerated by obesity-associated insulin resistance. In support of this notion, the candidate genes located in this region include β2-adrenergic receptor (*ADRB2*), natriuretic peptide receptor C (*NRP3*) and islet-1 (*ISL1*). *ADRB2 *is involved in vasodilation [39] while *NRP3 *inhibits adenyl cyclase [40], both of which are implicated in blood pressure control. The other candidate, *ISL1*, is a transcription factor that regulates insulin gene expression in the pancreatic islets [41].

The 155 cM region of chromosome 7 was linked to both glucose and lipid factors (TC-LDLC and TG-HDLC). Of note, nearby regions at 109-123 cM and 134 cM have been linked to low HDLC and visceral fat in other populations [30]. Ectopic especially visceral fat plays pivotal role in insulin resistance mainly through increased production of free fatty acids and cytokines. Insulin resistance in turn can lead to non-suppression of lipolysis and increased hepatic glucose production leading to hyperglycemia and dyslipidemia as components of the MES [42].

In this analysis, we identified six factors during factor analysis whereas other studies reported three to four factors only [22,23,43]. In the latter case, the insulin variables are commonly loaded on the same factor as adiposity variables. Our seemingly discrepant results might be due to population differences. For example, the study populations reported by Tang et al. [23] and Arya et al. [22] were substantially more obese (mean BMI = 27.6-28 and 29.1 kg/m^2^, respectively) than our cohort (BMI = 23.8 kg/m^2^). Since hyperinsulinemia and insulin resistance are mainly found in obese subjects, correlation between obesity and insulin resistance is likely to be more robust in an obese than a relatively lean population, such as Chinese.

Moreover, the nature and number of metabolic variables used to generate composite factors of the MES vary substantially across studies. While Bossé [43] and Arya [22] included eight MES-related variables, we included fifteen variables in our analysis. In the former studies [43,22], only TG and HDLC were used as the lipid variables and no oral glucose tolerance test (OGTT) data were presented. By contrast, we included four variables (TC, TG, LDLC and HDLC) to quantify dyslipidemia and included AUC for PG and PI during OGTT at 0-30 min and 0-120 min for insulin secretion. This comprehensive phenotypic dataset allows us to identify two blood lipid factors and segregate insulin resistance factor into its glucose and insulin components, which can have different biological determinants.

There are several limitations to this study. Firstly, we examined normoglycemic subjects only in order to exclude the impact of diabetes and medications on the measures of metabolic traits. Although such exclusion may reduce sample size and study power, our results in this subgroup analysis accord with our previously reported linkages to type 2 diabetes and individual MES-related traits in 1q21-25 regions [20,21], along with reports from other populations [33]. Secondly, in the era of genome-wide association studies where reproducible common genetic variants are successfully identified in complex diseases, there are fewer successful examples for discovering complex disease genes using the linkage approach alone [44,45]. However, linkage results are complementary and can be used to prioritize the linkage regions for investigation and follow up in genome-wide association and resequencing studies. This is exemplified by the discovery of TCF7L2 gene with type 2 diabetes [44,46]. Indeed, genome-wide linkage approach remains essential until high throughput technology that allows association analysis of both rare and common variants at a affordable cost becomes available [47].

## Conclusions

In conclusion, in a family-based cohort of normoglycemic subjects ascertained through probands with predominantly young onset diabetes, we found suggestive linkages to adiposity, insulin, glucose and lipid factors on chromosomes 1, 2, 5, 7, 9, 12, 13 and 17. Some of these regions overlap or in close proximity to other genome scans of MES traits. With increasing understanding of the pathogenesis of MES and related traits, exploration of these regions and their interactions, including epigenetics, will provide important insights into the nature of these interacting pathways and their relationships with clinical presentations, thereby bringing personalized diagnosis and therapy closer to reality.

## Methods

### Subjects

The study design, ascertainment, inclusion criteria and phenotyping of the HKFDS have been described elsewhere [20]. Briefly, 478 normoglycemic individuals (42% men) were selected from 163 families consisting of siblings, parents, spouses, and offspring (>16 years) ascertained through a proband with T2D. Patients with clinical or autoimmune type 1 diabetes and families with known maturity-onset diabetes of the young or mitochondrial DNA nucleotide 3243 A > G mutations were excluded. All first degree relatives underwent extended phenotyping including 75 gram OGTT and had complete phenotypic data for 15 MES traits. The average family size was 3 (2 - 4) (median (interquartile range)) members. Written informed consent was obtained from all participating subjects. This study was approved by the Clinical Research Ethics Committee of the Chinese University of Hong Kong.

### Clinical studies

Using a standardized protocol, all family members were examined after at least 8 hours of overnight fast. They underwent full clinical examination and completed a questionnaire on personal, family, medical and lifestyle histories. Anthropometric parameters including body weight and height, WC and HC, SBP and DBP were measured. Fasting blood samples were collected for measurement of PG, PI, lipid profile (TC, TG, HDLC and LDLC) and DNA extraction. AUC for PG and PI at OGTT 0-30 min, 0-120 min were calculated using the trapezoid rule.

### Genotyping

Detailed information on PCR conditions, genotyping procedure and quality control have been described [20]. Briefly, we made use of the Human Screening Set, version 10 for the previous genome scans. A total of 355 microsatellite markers (Research Genetics, Huntsville, AL) were genotyped across all 22 autosomes. The average spacing between markers was 10 cM, and average heterozygosity was 71%. In this analysis, 70 additional microsatellite markers were genotyped at chromosomes 1, 2, 4, 5, 6, 10, 12 and 16 to delineate whether the linkage signals observed in our previous linkage studies for diabetes, MES and its components [20,21] affect single or multiple metabolic factors. Genetic relationships among family members were checked by programs RELPAIR [48] and PREST (Pedigree Relationship statistical Test) [49], and corrected. Mendelian errors and potential genotyping errors were checked by PEDCHECK (version 1.1) [50] and MERLIN (version 1.01) [51], respectively, and removed.

### Statistical analysis

Data were transformed using natural logarithm (BMI, WC, HIP, SBP, DBP, TC, TG, fasting PG, fasting PI, PG AUC for 0 - 30 min and PG AUC for 0 - 120 min), or square root (PI AUC for 0 - 30 minutes, PI AUC for 0 - 120 minutes, HDLC and LDLC) due to skewed distributions. Outliers (<0.3%) with values greater than or equal to 4 standard deviations from the mean were removed. Data were then standardized to zero mean and unit variance by multiple linear regression models with age and sex as covariates.

In the factor analysis model, the amount of variance contributed by each factor was evaluated by eigenvalues, which represent the sum of the squared factor loadings. In the present study, factors with eigenvalues ≥1.0 were extracted. We then applied varimax rotation method to achieve a simple structure which showed improved interpretability of factors that were consistent with the underlying biological processes. Traits with factor loadings ≥0.40 in absolute value were used to interpret and characterize the factor structures. Finally, a factor score, which was the estimated values of underlying factor, was calculated for each individual based on the factor loadings and the values of observed phenotypes [23]. The factor analysis (using the PROC FACTOR procedure) and other statistical analyses were performed in SAS v.9.1 (SAS Institute, Cary, NC, USA) unless specified otherwise.

Multipoint variance components linkage analyses implemented in SOLAR v.2.0 [52] were conducted on the factor scores obtained from PCFA to identify linkage for each factor. Estimated heritability for each factor was also reported which estimated the proportion of total variance attributed by the additive genetic variance. To estimate our evidences of linkages, empirical p-values were obtained through the simulation of 10,000 replications of a fully informative marker under the null hypothesis of no linkage between marker and phenotype using SOLAR. A LOD score ≥3.0 was considered as significant evidence of linkage.

## Authors' contributions

CT performed the statistical analysis and interpretation of data, and participated in drafting the manuscript. VL helped to perform the analysis and participated in coordination and acquisition of data. WS, RM, JC and MN conceived of the study, and participated in its design and coordination and helped to draft the manuscript. All authors read and approved the final manuscript.

## Supplementary Material

Additional file 1 **Contains supplementary table 1** - Comparisons of multipoint linkage analyses results for other metabolic factors and its individual components. Click here for file

Additional file 2 **Contains supplementary table 2** - Supplementary Table 2: Summary of linkage findings in the present and other previous studies. Click here for file
