# Supplementary material for: Genome-wide linkage scan for factors of metabolic syndrome in a Chinese population
Source: BMC Genet. 2010 Feb 24;11:14. doi: 10.1186/1471-2156-11-14 (PMC2838753; doi:10.1186/1471-2156-11-14)
Supplement: Additional file 1 — Contains supplementary table 1 - Comparisons of multipoint linkage analyses results for other metabolic factors and its individual components. [file 1471-2156-11-14-S1.DOC]

Supplementary Table 1: Comparisons of multipoint linkage analyses results for other metabolic factors and its individual components.

|  | **Adiposity**  **Factor** | | | **Insulin**  **Factor** | | | **Glucose Factor** | **TCLDL**  **Factor** | | **TGHDL Factor** |
| --- | --- | --- | --- | --- | --- | --- | --- | --- | --- | --- |
| **Chromosome (Position [cM])** | **1 (187)** | **9 (34)** | **17 (10)** | **2 (128)** | **5 (21)** | **12 (7)** | **7 (155)** | **7 (151)** | **13 (15)** | **7 (155)** |
| **LOD** | 2.22 | 1.92 | 2.46 | 2.23 | 1.61 | 1.92 | 2.16 | 1.24 | 1.43 | 1.96 |
| **Empirical P value** | 0.0022 | 0.0038 | 0.0013 | 0.0018 | 0.007 | 0.0037 | 0.0003 | 0.009 | 0.0064 | 0.002 |
| **Body mass index (kg/m^2^)** | 1.58 | 1.23 | 2.83 |  |  |  |  |  |  |  |
| **Waist circumference (cm)** | 1.57 | 1.59 | 2.14 |  |  |  |  |  |  |  |
| **Hip circumference (cm)** | 1.30 | 1.23 | 2.21 |  |  |  |  |  |  |  |
| **Fasting plasma Insulin (pmol/l)** |  |  |  | 0.03 | 0 | 0.20 |  |  |  |  |
| **Insulin AUC 0 – 30 minutes (pmol/l)** |  |  |  | 3.52 | 0.95 | 1.51 |  |  |  |  |
| **Insulin AUC 0 – 120 minutes (pmol/l)** |  |  |  | 0.12 | 0.86 | 2.31 |  |  |  |  |
| **Fasting plasma glucose (mmol/l)** |  |  |  |  |  |  | 0.66 |  |  |  |
| **Plasma glucose AUC 0 – 30 min (mmol/l)** |  |  |  |  |  |  | 0.57 |  |  |  |
| **Plasms glucose AUC 0 – 120 min (mmol/l)** |  |  |  |  |  |  | 1.53 |  |  |  |
| **Total cholesterol (mmol/l)** |  |  |  |  |  |  |  | 1.25 | 1.75 |  |
| **LDL cholesterol (mmol/l)** |  |  |  |  |  |  |  | 1.56 | 0.65 |  |
| **Triglyceride (mmol/l)** |  |  |  |  |  |  |  |  |  | 2.16 |
| **HDL cholesterol (mmol/l)** |  |  |  |  |  |  |  |  |  | 0.94 |
