# Supplementary material for: Genome-wide linkage scan for factors of metabolic syndrome in a Chinese population
Source: BMC Genet. 2010 Feb 24;11:14. doi: 10.1186/1471-2156-11-14 (PMC2838753; doi:10.1186/1471-2156-11-14)
Supplement: Additional file 2 — Contains supplementary table 2 - Supplementary Table 2: Summary of linkage findings in the present and other previous studies. [file 1471-2156-11-14-S2.DOC]

**Supplementary Table 2: Summary of linkage findings in the present and other previous studies.**

| **Study [reference number]** | **Metabolic traits** | **Chr** | **Position (cM)** | **No. of family** | **N** | **LOD** |
| --- | --- | --- | --- | --- | --- | --- |
| *Present study* | *Adiposity* | *1* | *187* | *163* | *478* | *2.22* |
| Ng *et al*., 2004 [20] | Metabolic syndrome | 1 | 169.5-181.5 | 179 | 897 | 4.5 |
| Ng *et al*., 2004 [21] | Type 2 diabetes | 1 | 173.9 | 64 | 401 | 3.09 |
| Arya *et al.* 2002 [22] | Adiposity-insulin factor | 1 | 188 | 27 | 261 | 2.6 |
| Arya *et al.* 2002 [22] | Lipid profile factor | 1 | 217 | 27 | 261 | 1.9 |
| Jacobson *et al*., 2006 [36] | Resting metabolic rate | 1 | 1q21.2 | 169 | 426 | 2.44 |
| Das *et al*, 2007 [38] | Diabetes | 1 | 1q21-25 | -- | -- | -- |
| Bossé *et al.*, 2007 [52] | Metabolic syndrome factor | 1 | 1p | 264 | 707 | > 1.75 |
| *Present study* | *Insulin* | *2* | *128* | *163* | *478* | *2.23* |
| Arya *et al.* 2002 [22] | Adiposity-insulin factor | 2 | 162 | 27 | 261 | 2.3 |
| Tang *et al.*, 2003 [23] | Metabolic syndrome factor | 2 | 240 | 1244 | 5975 | 3.34 |
| von Wowern *et al*., 2003 [30] | Young onset hypertension | 2 | 109-121 | 91 | 426 | 1.4-1.8 |
| Liu *et al*., 2004 [31] | Hypertension | 2 | 83.4-111.8 | -- | 6245 | -- |
| Perusse *et al*., 2001 [35] | abdominal subcutaneous fat | 2 | 102 | 156 | 521 | -- |
| Perusse *et al*., 2001 [35] | abdominal subcutaneous fat | 2 | 168 | 156 | 521 | -- |
| Palmer *et al*., 2003 [37] | BMI | 2 | 149 | 66 | 349 | 1.72 |
| Kalmyrzaev *et al*., 2006 [41] | Premature hypertension | 2 | 166-176 | 1 | 44 | 2.68 |
| *Present study* | *Insulin* | *5* | *21* | *163* | *478* | *1.61* |
| Freedman *et al*., 2005 [33] | Insulin | 5 | 20 | 805 | 2589 | 2.36 |
| Palmer *et al*., 2003 [37] | BMI | 5 | 14 | 66 | 349 | 1.81 |
| Bell *et al*., 2006 [42] | Hypertension | 5 | 5q13.3, 5q14 | 1639 | 2076 | -- |
| *Present study* | *Glucose* | *7* | *155* | *163* | *478* | *2.16* |
| *Present study* | *TC-LDL-C* | *7* | *151* | *163* | *478* | *1.24* |
| *Present study* | *TG-HDL-C* | *7* | *155* | *163* | *478* | *1.96* |
| Arya *et al.* 2002 [22] | Lipid profile factor | 7 | 130 | 27 | 261 | 3.2 |
| Arya *et al.* 2002 [22] | Lipid profile factor | 7 | 178 | 27 | 261 | 1.9 |
| Tang *et al.*, 2003 [23] | Metabolic syndrome factor | 7 | 135 | 1244 | 5975 | 2.42 |
| Perusse *et al*., 2001 [35] | abdominal visceral fat | 7 | 134 | 156 | 521 | 1.97 |
| Palmer *et al*., 2003 [37] | BMI | 7 | 175 | 66 | 349 | 1.65 |
| Arya *et al*., 2002 [40] | HDL-C | 7 | 109-123 | 27 | 415 | 1.6 |
| Bossé *et al.*, 2007 [52] | Metabolic syndrome factor | 7 | 7p | 264 | 707 | > 1.75 |
| *Present study* | *Adiposity* | *9* | *34* | *163* | *478* | *1.92* |
| Arya *et al.* 2002 [22] | Lipid profile factor | 9 | 28 | 27 | 261 | 2.0 |
| Arya *et al.* 2002 [22] | Adiposity-insulin factor | 9 | 67 | 27 | 261 | 2.8 |
| Liu *et al*., 2004 [31] | Hypertension | 9 | 32.5-59.8 | -- | 6245 | -- |
| Badzioch *et al*., 2004 [34] | familial dyslipidemia | 9 | 24 | 4 | 185 | 3.7 |
| Perusse *et al*., 2001 [35] | visceral fat area, HDL-C | 9 | 26.8 | 156 | 521 | -- |
| Arya *et al*., 2002 [40] | Type 2 diabetes | 9 | 25 | 27 | 415 | 2.4 |
| Arya *et al*., 2002 [40] | HDL-C | 9 | 41 | 27 | 415 | 3.4 |
| *Present study* | *Insulin* | *12* | *7* | *163* | *478* | *1.92* |
| Arya *et al.* 2002 [22] | Adiposity-insulin factor | 12 | 119 | 27 | 261 | 2.2 |
| Tang *et al.*, 2003 [23] | Metabolic syndrome factor | 12 | 83 | 1244 | 5975 | 2.86 |
| Palmer *et al*., 2003 [37] | Apnea-hypopnea index | 12 | 7 | 66 | 349 | 1.43 |
| Palmer *et al*., 2003 [37] | BMI | 12 | 14 | 66 | 349 | 1.73 |
| *Present study* | *Adiposity* | *17* | *10* | *163* | *478* | *2.46* |
| Rich *et al*., 2005 [32] | Fasting insulin, fasting glucose | 17 | 54 | 132 | 1604 | 3.3 |
| Palmer *et al*., 2003 [37] | Apnea-hypopnea index | 17 | 1 | 66 | 349 | 1.11 |
| Palmer *et al*., 2003 [37] | BMI | 17 | 22 | 66 | 349 | 1.04 |
| Demenais *et al*., 2003 [39] | Type 2 diabetes | 17 | 29-58 | -- | 3947 | 1.54 |
